# Scanning Tunneling Microscopy of Biological Structures: An Elusive Goal for Many Years

**DOI:** 10.3390/nano12173013

**Published:** 2022-08-31

**Authors:** Andrés Rodríguez-Galván, Flavio F. Contreras-Torres

**Affiliations:** 1 Carrera de Biología, Unidad de Biomedicina, Facultad de Estudios Superiores Iztacala, Universidad Nacional Autónoma de México, Tlalnepantla 54090, Edo. Mex., Mexico; 2 Tecnologico de Monterrey, The Institute for Obesity Research, Monterrey 64849, NL, Mexico

**Keywords:** scanning tunneling microscopy, nucleic acids, carbohydrates, proteins, lipids

## Abstract

Scanning tunneling microscopy (STM) is a technique that can be used to directly observe individual biomolecules at near-molecular scale. Within this framework, STM is of crucial significance because of its role in the structural analysis, the understanding the imaging formation, and the development of relative techniques. Four decades after its invention, it is pertinent to ask how much of the early dream has come true. In this study, we aim to overview different analyses for DNA, lipids, proteins, and carbohydrates. The relevance of STM imaging is exhibited as an opportunity to assist measurements and biomolecular identification in nanobiotechnology, nanomedicine, biosensing, and other cutting-edge applications. We believe STM research is still an entire science research ecosystem for joining several areas of expertise towards a goal settlement that has been elusive for many years.

## 1. Introduction

Biomolecules are chemical structures produced by living organisms that primarily carry out essential functionalities such as catalysis, generation and transmission of signals, and immune defense, thus taking an essential role in the metabolism and reproduction of living organisms. The most critical aspect of biomolecules is their fundamental relationship between structure and function. DNA stores genetic information about the composition of proteins. RNA is typically a single-stranded biopolymer responsible for biochemical reactions, like enzymes, mainly in cellular protein synthesis. Proteins are macrostructures consisting of a typical sequence of amino acids that ultimately determine different functions. Lipids and carbohydrates have structural roles and participate in cell signaling and as a source and reservoir for energy. Unlike organic engineered structures prepared in laboratories, biological structures are dynamic and self-organize; they sculpt themselves and change their architecture. Biological structures have structural building blocks that generate force and constantly come on and off [1].

The structure and function of biomolecules can be influenced by environment conditions where biomolecules occur. Therefore, the structural characterization, the identification of physical interactions, and the direct imaging of biomolecules are of great value to a better understanding of the functionalities of biomolecules in different environments. In particular, the visualization of biomolecules also aims at supporting the rational design of new complexes or customized substances with specific properties. X-ray crystallography [2], nuclear resonance spectroscopy (NMR) [3], and transmission electron microscopy (TEM) [4] are alternative or complementary techniques in identifying the structure of biomolecules. However, in the case of X-ray techniques, several experimental conditions such as efficient protein extraction, solubilization, stabilization, and generating diffracting crystals for some proteins (i.e., membrane proteins) can limit their performance. More importantly, the information obtained only refers to the average configuration of the molecules inside the crystal and not the information of individual biomolecules [5]. The analyses using NMR require higher concentrations of biomolecules. The samples need to be stabilized for long periods, avoiding aggregation because NMR usually takes several days for the data acquisition (i.e., 3D HNCO) [6]. Finally, biomolecules can be challenging to visualize using TEM. Sometimes, the electron current required to achieve an acceptable signal-to-noise ratio can damage the biomolecules. Additionally, heavy metals for staining can give inaccurate structural information; they are highly mobile under high-energy electron beams, which leads to fuzzy images [7].

Scanning tunneling microscopy (STM) is a technique that allows the visualization of atoms at solid surfaces [8]. STM was created as a method for the analysis of conductive surfaces; however, a time after their development, it was reported that biological samples could be analyzed by STM when deposited on conductive surfaces [9]. The interest in using STM for biological characterization traces back to the late 1980s. Former reports suggested that STM could be operated underwater and with other fluids [10]. In this way, it was thought that STM could be used to visualize biological molecules in conditions that can mimic those found in vivo. STM could analyze biomolecules in a liquid environment, allowing the measurements in the native occurrence. STM can also provide structural information without significant sample preparation procedures contrary to TEM (i.e., avoiding fixation and staining). A few micrograms are required for the analysis, and usually, samples can be preserved for further analysis. Furthermore, STM showed the capability to resolve assemblies on flat surfaces or when biomolecules formed larger complexes. This technique is not limited to analyzing only small individual biomolecules but also fragments of assemblies [11,12]. The potential for determining the structures of biomolecules in aqueous environments emphasizes the importance of STM in biology, technology, and instrumentation. STM and atomic force microscopy (AFM) are part of the family of scanning probe microscopes (SPM) that are characterized by using a very sharp tip to sense the surface of a sample [8]. However, AFM is not restricted to conductive samples, and it is currently most often used for imaging biomolecules [13]. AFM can image biological samples at the nanoscale. It can help to characterize mechanical properties, such as elasticity, viscosity, and adhesion [14]. The objective of STM is not to compete with X-ray crystallography, NMR, TEM, or AFM, which are much more suitable for high-resolution structures, but rather to use STM for systems in which other functional analyses are needed.

Herein, we focus on the reported applications of STM to study biological systems. Different studies on biomolecule measurements were scrutinized and organized (e.g., DNA, lipids, proteins, and carbohydrates). We first reviewed theories about STM imaging formation. Further, the relevance of STM imaging of biomolecules is presented by comparing the current state-of-the-technique in the field. Perspectives are manifested considering that the potential utility of STM has yet to be fully realized in several biological systems. Within this framework, this review resumes the investigations that continue appealing to STM to study the structure of biological samples. It is worth mentioning that STM imaging is operationally supported by developing instrumental accessories to operate in several conditions, from a vacuum to liquid, allowing it to mimic physiological environments.

## 2. STM Imaging Formation and Contrast Mechanisms

**Figure 1:**
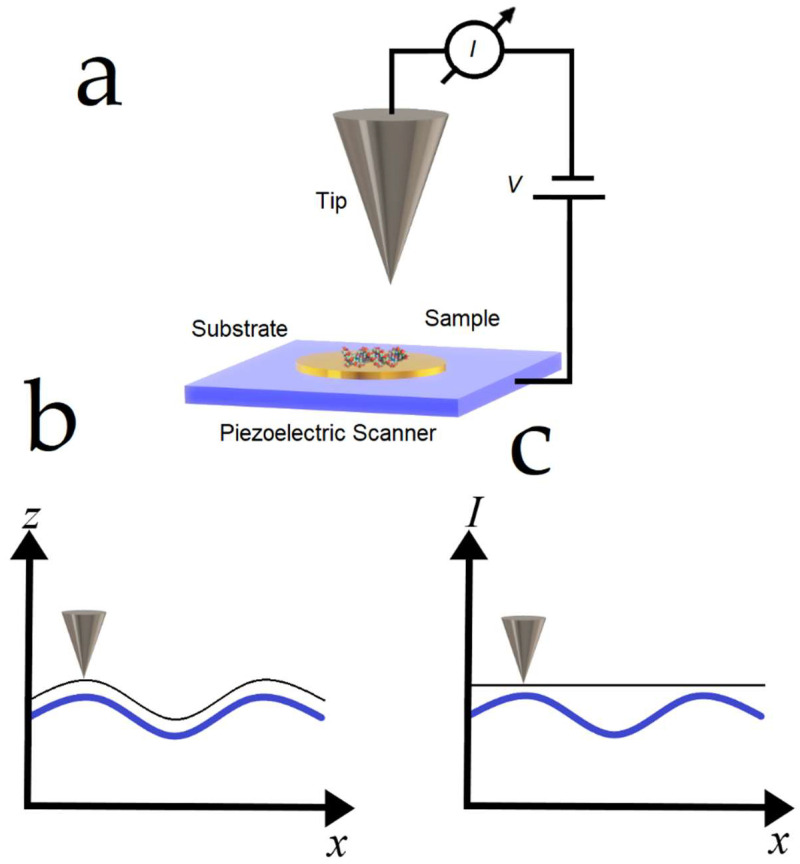
Schematic illustration of basic components of STM. STM collects the tunneling current between the tip apex and the sample when a bias voltage is applied (**a**). Relative, precise, lateral sample-lever movement is achieved by mounting either the sample or the tip on the end of a piezoelectric scanner. The tip is the physical sensing probe, a fine wire cut or etched to form a very sharp tip. The ideal tips have a single atom at the apex because they can provide images at atomic resolution. Generic STM operating modes in constant-current (**b**) and constant-height (**c**).

The principle of STM is illustrated in Figure 1. A typical STM consists of a system that conducts a current between a conductive tip and a conductive sample. Only at a few nanometers of the contacting regimen, the sample (conductive specimen) receives primary electrons through the “tunnel effect” ejected from a metallic tip (Figure 1a). The small tunneling current (10 pA to 1 nA) depends on the tip-surface distance and the bias voltage; therefore, these parameters can be optimized to scan the surface at high resolution [15]. A piezoelectric scanning unit controls the vertical and lateral movement of the tip. Hence, STM images are acquired by two different modes. The images can be generated for the constant current mode by adjusting the tip-sample distance (Figure 1b); otherwise, the image is generated through variations in the tunneling current in the constant height mode (Figure 1c). The constant height mode is preferably used in flat surfaces to avoid loss of current sensitivity. Alternatively, constant current mode is employed for samples with high roughness or protrusions that can damage the tip while scanning the surface. Finally, digital feedback collects the output signal from the current pre-amp, compares the signal level with the preset value, calculates the response according to the user-defined feedback parameters, and sends the feedback voltage. The feedback signal and tunneling current signal return to an electronic card in the computer to generate topographic images.

Because STM is based on the flow of an electrical current, the obtained images represent a convolution of the topography and electronic properties of the sample. Hence, STM cannot directly image insulating material. Only organic samples thinner than about 1 nm, which allow tunneling through the material, can be imaged with good results. Therefore, STM images of a nonconducting sample may not be interpreted as the surface topology. It is noteworthy that the interpretation of STM images is not straightforward; the resolved images usually do not reflect a natural topographic position of atoms into the molecular system but rather constitute the response from the electronic states contributing to the tunneling between tip and sample [16].

Many theories were proposed to explain the contrast formation in STM. In 1989, Spong et al. [17] suggested a contrast mechanism for resolving organic molecules with tunneling microscopy. The authors, revealed that STM could resolve the individual molecules and distinguish between the different functional groups within the molecules. The contrast mechanism responsible for the well-resolved images was proposed to be a modulation of the local work function of the substrate by the polarizable molecular adsorbates. Hence, contrast mechanisms for detecting individual segments in large molecules were related to the dependence of the local surface conductivity on the individual hydrophilicity of the molecular groups located directly under the tip. In 1990, Miles et al. [18] demonstrated that STM could be used to image a range of biological molecules. They reported studies on proteins and polypeptides in extended helical conformation; they presented images from a sample of ethidium bromide bound to linearized plasmid DNA; they also investigated α-cyclodextrin when adsorbed onto graphite substrate that appears to form lattices. It was suggested that the contrast mechanism is due to the dipole moment of the adsorbed molecule reducing the work function of the substrate (e.g., graphite). Subsequent studies suggested that STM can obtain images for non-conductive samples in humid air atmosphere, implying that the mechanism may be indirectly related to the physisorbed water molecules.

Several years ago, STM imaging analysis noted dependence on humidity. Gucken-berger et al. [19] carried out experimental STM measurements to show that one monolayer of water at the investigated surface is sufficient for successful STM imaging of insulating material at low current. Two types of experiments were performed: measuring the surface conductivity of bulk insulators as a function of the ambient air’s humidity and imaging such surfaces and several biological samples prepared on mica. These showed the dependence of the steady-state current on ambient humidity. The average thickness of the water layer on a mica surface depends on the ambient air’s relative humidity (RH), which ranges from 0.15 nm at 45% RH to 0.4 nm at 70% RH. Hence, it was proposed that such a monolayer acts as a conductive coating, similar to the metal coating commonly used for insulating materials for scanning electron microscopy (SEM) [19]. Therefore, a monolayer film of water covering the sample can explain STM imaging formation of electrically insulating biomolecules such as proteins (with resistivity typically in about 10^15^–10^18^ Ωm). This explication can be supported by experimental evidence of the electrical conductance observed in hydrated proteins [20]. Typically, the wool can show a semiconductive behavior in a hydrated state. Similarly, the conductivity of crystalline bovine hemoglobin increases when it is hydrated [21]. These observations suggest that thicknesses of nonconducting materials are limited to very thin preparations in the range of just a few nanometers.

Another question concerns the gap between the tip and the biological sample. Water molecules might bridge this distance as a mechanism for imaging biological material [22]. Assuming that the thin film of water is connected with an infinitely large reservoir to ensure a moist surface on the sample, the formation of the water bridge can validate the idea that the water molecules provide a closed circuit for ion conduction. According to the authors [22], this expectation is incorrect because this idea has implicitly assumed that the tip surface is dry around the contact area. The tip materials are not easy to wet due to cleaning before measurements; a tip with a small radius is difficult to wet. They concluded that the apical region of the tip should be dry at every moment, even if the whole tip is wet. The tip oscillated at higher humidity, while no images could be obtained at lower humidity. There is a possibility that the critical radius of the tip becomes small enough when humidity is sufficiently high; in this way, the water film merges with the bridge on the tip, and the contact originates the current. Nevertheless, the STM tip is a macroscopic object that is rarely in equilibrium with the surface. The exact geometry of the tip is commonly unknown except for some outstanding STM measurements, where the tip structure was determined before and after a scan by field-ion microscopy [23].

Ultra-high vacuum (UHV) can assist in obtaining images of individual atoms and determining energy states on an atom-by-atom basis [10]. At such conditions, the tunneling current is an exponentially decaying function of the separation between the tip and the surface. This fact enhances the tunneling, creating contiguous segments of the conductively coated surface, mainly using platinum/carbon (Pt/C). Thus, the coated segments of biological samples are conductive and mechanically stable to allow easy identification by STM. This method has been primarily reported for DNA imaging, commonly deposited on HOPG and mica substrates. It was reported that the micrometer-sized conductive regions created by masking might be influenced by the state of hydration of the molecules, which may alter their conductivity and change their contrast [24]. The analysis of biological samples in UHV conditions shows reproducibility in the terminus of macromolecular structure. So far, UHV maintains the samples free of temperature changes and free contaminants.

The mechanism for contrast formation for non-conductive samples remains unsuccessfully understood. The difficulty relies on the nature of the phenomenon. The tunneling junction is transported from a metal tip towards the substrate and through the sample (non-conductive). Generally, for small organic molecules, it is needed to immobilize the samples to achieve atomic resolution. Since the current in the tunneling junction is always due to the same physical process, this process is determined by (i) the distance between the leads, (ii) the chemical composition of surface and tip, (iii) the electronic structure of both systems, (iv) the chemical interactions between surface and tip atoms, and (v) the electrostatic interactions of sample and tip [16]. Hypothetically, a single atom at the apex of clean and sharp tips is ideal for providing atomic resolution. However, it is not feasible in experiments due to salts, surfactants, or biological impurities in the solutions where biological samples are analyzed, which usually contaminate the tip or promote flattening that affects the imaging resolution [25].

The poor electron conductivity of most biological samples is another factor that restricts the application of STM for imaging biological materials. Hence, the development of substrates to allocate the biological samples has facilitated the preparation and identification of DNA, lipids, and proteins (see Section 3). In this regard, metal substrates and HOPG are commonly chosen to attach biological samples. It should be noted that the presence of defects which can mimic biological samples in HOPG, promote the denaturation of proteins because of their hydrophobic nature [26]. Gold substrates can be a more feasible option to attach proteins because of the strong interactions; thiol-functionalized Au substrates favor image acquisition and decrease the mobility of the samples. The methodology employed for the deposition of samples commonly uses drop casting [27]. This deposition way is easy and rapid for sample preparation despite its limitations related to the lack of ability to control uniformity deposits [28]. For instance, areas of high concentration can be difficult to scan because the tip is usually prone to contamination or to disrupt the fragile structure of biomolecules. It has been suggested that well-dispersed thin samples consisting of a single layer of biomolecules are critical in achieving good reproducibility [8]. Finally, the electrospray ion beam deposition (ES-IBD) is a technology that can be key and necessary to immobilize molecules aiming for reproducible images. ES-IBD is a proven technique for depositing carbohydrates, peptides, and proteins without damaging the integrity of biomolecules [29].

The most significant challenge and a primary goal of STM, AFM, and other surface microscopies is the single atoms imaging within a molecule. Several relationships and differences between STM and AFM have been proposed for several decades. In particular, the existence of a direct correlation between atomic forces and tunneling has been the most significant paradigm among these two techniques. On the one hand, atomic forces are subject to pure mechanical measurements. The short-range forces can be repulsive (Pauli) or attractive (van der Waals and electrostatic forces). On the other hand, tunneling is subject to pure electrical measurements. The tunneling current in STM is sensitive to the local electron density of states close to the Fermi level. The coexistence of tunneling conductance and attractive atomic forces was first discovered by Dürig, Züger, and Pohl in 1988 [30,31]. It has been demonstrated that as a consequence of the Schrödinger equation, tunneling and covalent bonding are equivalent. It was found that the attractive force varies exponentially with tip-sample separation, with a decay length roughly twice the decay length of tunneling conductance. Alternatively, tunneling conductance is proportional to the square of covalent-bond energy. The tunneling conductance varies with tip-sample separation by a decay length of about 0.043 nm. The force varies with such separation by a decay length of about 0.086 nm. This fact represents the equivalence between tunneling and interaction energy for two interacting systems. Today, the principle of reciprocity is well established and verified in terms of STM [32]. Several experiments have confirmed such equivalence [33,34,35,36].

The atomic resolution has also been achieved in several AFM investigations [36]. Gross et al. [37] demonstrated that modifications of the AFM tip lead to dramatically enhanced atomic-scale contrast. They showed that the exact atomic composition and the geometry of the tip are the main factors influencing the atomic resolution. It is necessary to operate in the short-range forces regime where chemical interactions result in substantial contributions for imaging. Operationally, STM imaging at the atomic scale is more challenging because the tunneling current is sensitive to the local electron density of states. STM can image features on the molecular surface, but resolving single atoms within an adsorbed molecule can lead to misleading interpretations. For instance, STM images the molecular orbitals near the Fermi level when molecules are adsorbed onto insulating films. However, STM produces broadened and distorted molecular orbital when molecules are adsorbed on metals. The latter is due to coupling to the electronic states of the substrate and molecules where an energetic coupling of two parties, or two atomic orbitals, can form a weak adhesion (about 10–16 J). In this way, STM is sensitive to substrate choices, whereas AFM images are more sensitive to the tip’s functionalization and geometry.

## 3. Imaging of Biomolecules by STM

### 3.1. DNA

One of the first biomolecules studied by STM was double-stranded DNA. Images underwater and air have been reported. DNA is relevant since all living organisms use it to store information to develop, survive, and reproduce [38]. DNA is constituted by two paired strands complementary in their nucleotide sequence and assembled in a double helix. The model for DNA double helix was proposed in 1953 by James Watson and Francis Crick based on Rosalind Franklin’s and Maurice Wilkin’s X-ray diffraction patterns. Since discovering the double helix, several attempts have been focused on the direct imaging of DNA; however, the challenge remains elusive; some works by HRTEM indicate it is difficult to resolve DNA structure because of the low signal-to-noise ratio (SNR) imaging [4].

As mentioned, DNA is among the first biomolecules visualized by STM. In 1987, DNA samples were incubated with the recA-protein to form a complex, fixed with glutaraldehyde, and purified to eliminate the unbound protein [39]. The samples were deposited on highly oriented pyrolytic graphite (HOPG). STM experiments were performed at air and room temperature (RT). The images obtained were difficult to interpret; however, the authors showed that meaningful structural imaging by STM is possible on bare biological samples. Years later, Driscoll et al. [40] reported the atomic-resolution imaging of duplex DNA by STM in an ultra-high vacuum and using freshly cleaved HOPG as substrate. They reported a correlation between the observed STM profiles with data derived from X-ray crystallography of DNA in the A-form. It was resolved in both the major and minor grooves of the double helix.

Those first reports showed the capability of STM for imaging DNA. However, skepticism emerged when it was reported that bare HOPG often exhibits defects similar to DNA fragments. Hence, one must be cautious when analyzing biological samples deposited on HOPG. However, such local defects on HOPPG are very stable to significant variations of imaging parameters; thus, this characteristic can be used to identify biological samples [41].

**Figure 2:**
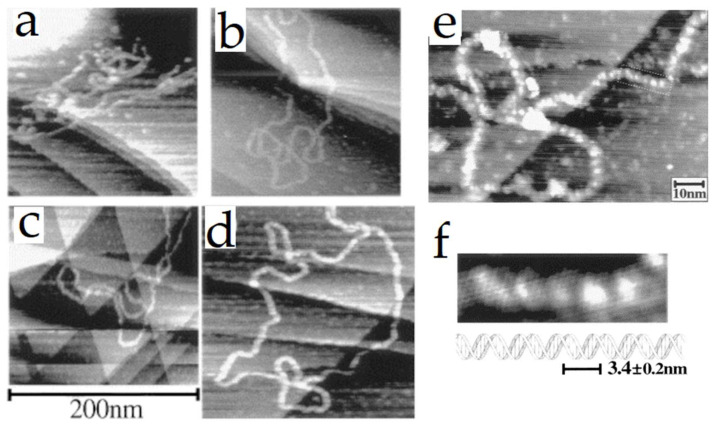
STM images of 2739 base-paired double-stranded plasmid DNA molecules deposited on a Cu (111) surface. The samples were deposited using the pulse injection method. Typical STM images of plasmids (**a**–**d**). A magnified image of a fragment of (**d**) is shown in (**e**) and a high-resolution image of a selected area of (**e**) shows a periodic structure with a periodicity of 2.6–3.6 nm along the strand is visible (**f**). For comparison, one scheme of DNA at the same scale is included. Reprinted with permission from Ref. [42]. Copyright (1999), Elsevier.

Because of the concerns found with HOPG, the use of alternative substrates was explored for the analysis of DNA. For example, Tanaka et al. [42] used Cu(111) as substrate (Figure 2). They analyzed short single-stranded and double-stranded plasmid DNA (of 3 k base-pairs) under ultrahigh vacuum (UHV) conditions. The images exhibited the detailed structure of DNA. Some short single-stranded DNA molecules formed a double helix with a pitch of helical periodicity ~3.5 nm and a height of 0.16–0.36 nm. Values that correspond with the values of Watson–Crick model for DNA in its B-form (~3.4 nm and ~2 nm, respectively). The double-strand exhibited a topographic height of 0.2–0.5 nm and a strand diameter of about 2–4 nm, and an internal periodicity of 2.6–3.7 nm along. These values were near the Watson–Crick DNA model; the authors explain the slight discrepancy in periodicity may be due to the interaction of DNA with the substrate and the high flexibility of DNA chain [42].

**Figure 3:**
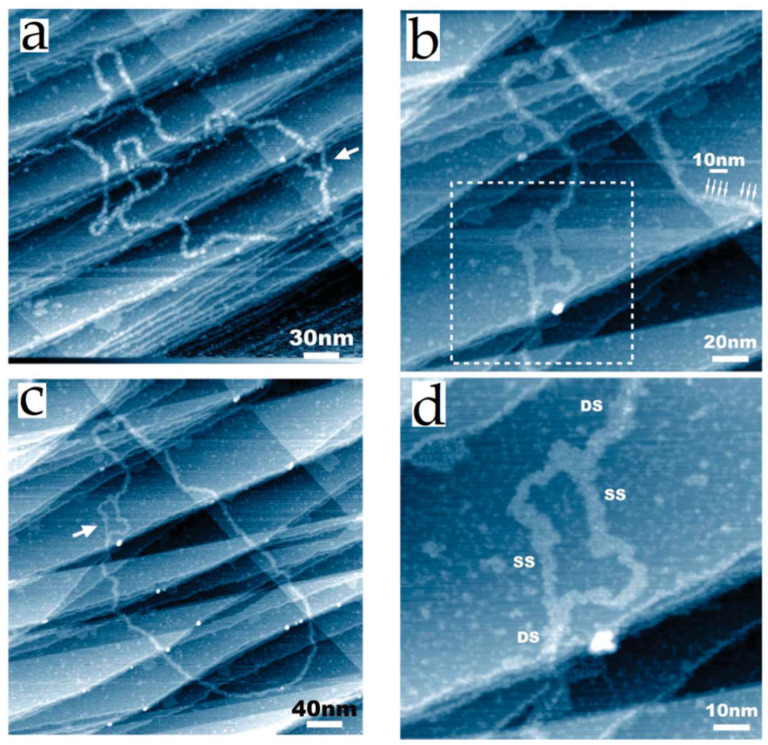
STM images of 2871 base-paired plasmid DNA were deposited on a Cu (111) surface using a pulse injection method. Typical STM images of plasmids (**a**,**c**). A higher magnification image of the area indicated with a white arrow in (**c**) is shown in (**b**). The white dotted square region of (**b**) shows a bubble-like structure, where single strands are indicated as SS, and double-stranded segments are indicated as DS (**d**). Reprinted with permission from Ref. [48]. Copyright (2010), American Chemical Society.

One of the main advantages of this work was the method of deposition. They used the pulse injection method under UHV conditions, avoiding sample degradation [43]. Gold surfaces have also been explored as a substrate for DNA analysis. Jing et al. [44] analyzed several synthetic oligonucleotides adsorbed on Au (111) electrode, and Wang et al. [45] examined single-stranded DNA (ssDNA) from denatured DNA plasmids using gold as substrate. More recently, Shapir et al. [46] analyzed poly(G)–poly(C) DNA molecules of 4000 base pairs long deposited on gold substrates. This group explored the electrostatic deposition method. The gold surface was positively biased (0.18 V) during the incubation of DNA solution on the substrate. The analysis was performed at room temperature and ultra-high vacuum conditions. STM images showed DNA structures like ropes that were clearly distinguished from the substrate. The height of the ropes was around 0.8 nm, and it showed a globular pattern along the molecule. The longitudinal profile showed an average length of ∼4 nm, slightly longer than expected for the B-form of DNA (3.4 nm). The authors suggested this discrepancy could be because poly(G)−poly(C) DNA adopts the A-form instead of the B-form; the A-form can be adopted by native DNA in low humidity conditions [47]. The use of metals as substrates for analyses of DNA has permitted overcoming the artifacts observed in HOPG. The reported images have displayed consistency in the macromolecular structure of DNA. They have helped differentiate between single and double-stranded DNA in an intact plasmid [48]. The single-stranded chains seemed more meandering and lower in apparent topographic height than double-helical regions (Figure 3). Additionally, the development of efficient deposition techniques has shown that it is possible to identify guanine base molecules in extended and fixed single DNA chains (ssDNA). The individual nucleotides were observed as bright points along the single chain. Some points were brighter than others along the chain. These brightest points were assigned as guanines by correlating imaging and spectroscopic data of oligomers containing a remarkable number of guanine nucleotides. More interesting, when STM images were aligned with part of the known base sequence of the analyzed ssDNA, the brightest point matched with the position of the guanines in the sequence [49]. These results opened the possibility of simultaneous sequencing and structural mapping of individual DNA molecules [50].

As was mentioned, STM can be used for the study of hybrid materials. Recently, Fardian-Melamed et al. [51,52] analyzed nanowires consisting of silver-containing poly(dG)–poly(dC) DNA molecules. STM allowed the detailed structural analysis of their morphological characteristics and electronic properties. Those nanowires are envisioned for applications in diverse areas such as nanolithography, energy conversion and storage, catalysis, sensing, and biomedical engineering [53].

### 3.2. Lipids

Lipids are very diverse biomolecules in terms of structure; however, they share the property that they are insoluble in water. Lipids play several fundamental biological functions: energy storage, biological membranes, signal messengers, and molecular recognition [54]. Lipids are among the first biomolecules analyzed by STM. Smith et al. [55] reported the molecular structure of arachidic acid bilayers on graphite by STM in air. The samples were deposited with the Langmuir–Blodgett technique. They reported the observation in the order of the organization of the lipids. Hörber et al. [56] analyzed the assembly of di-myristoyl-phosphatic acid (DMPA) on graphite, oxidized graphite, and gold-coated calcite. The Langmuir–Blodgett technique also prepared the samples, and the analysis was performed in the air. They reported that the lipids formed tightly packed films, where embedded proteins could be retained and stable for imaging. Matsuda et al. [57] analyzed fatty acid molecules deposited by the Langmuir–Blodgett technique on highly oriented pyrolytic graphite and molybdenum disulfide. Interestingly, they observed that the lipids formed parallel arrangements when the analysis was performed in air conditions. Woodward et al. [58] explored the use of a modified freeze-fracture replication technique to overtake the poor conductivity of lipids and analyzed bilayers of phosphatidylcholine in the ripper phase form. They reported that the images obtained by STM presented a superior resolution than direct imaging by TEM. They mentioned the images were easier to interpret and reproduce [58]. In addition to fatty acids, complex structures such as liposomes were analyzed by STM. Zareýe et al. [59] analyzed cationic liposomes deposited on a mica covered with gold (111) in air conditions at room temperature. They reported that STM could be useful for analyzing fragile liposomal samples. The images were permitted to calculate the diameter of the liposomes.

More recently, STM was employed to study the formation of lipid bilayers on gold surfaces. The study of lipid bilayers by STM proposes surfaces for biosensing devices. It investigates biomembrane-related processes such as protein binding, interactions with peptides or enzymes, and transport phenomena. Xu et al. [60] analyzed the formation of bilayers by spreading small unilamellar vesicles (SUVs) of DMPC (1,2-dimyristoyl-sn-glycero-3-phosphatidylcholine) on Au(111) electrode surfaces. According to the as-obtained STM images, the DMPC molecules were adsorbed with the acyl chains oriented parallel to the surface and assembled into an ordered monolayer. With time, the molecules were reoriented, and the monolayers were transformed into hemimicellar films. Sek et al. [61] analyzed the assembly of suspensions of vesicles of DMPC/Cholesterol on Au(11) electrode surfaces. Remarkably, they used electrochemical STM (EC-STM) for their analysis. Under these conditions, the self-assembly process was controlled by the hydrocarbon skeleton−metal surface interaction and observed the formation of ordered domains of either pure DMPC or pure cholesterol. The formation of these domains was transient because when more molecules accumulate at the surface, the molecule−molecule interactions start to dominate. The monolayer is then transformed into a bilayer [61]. Pawłowski et al. [62] described a mechanism for the lipid bilayer formation on an Au(111) based on SUVs of DMPC/cholesterol. They explained that the molecules are adsorbed with flat-lying orientation and formed stripe-like domains. Then, the monolayers serve as a template for developing hemimicellar films; a single planar bilayer is formed due to the fusion between coupled layers. EC-STM facilitates the molecular resolution imaging of lipid films with embedded pores formed by gramicidin [63], alamethicin [64], and trichogen [65]. These studies revealed the differences in aggregation behavior of these peptides within lipid films. Gramicidin preferentially creates individual channels, whereas alamethicin and trichogyne tend to form clustered assemblies with the aggregation pattern of the barrel-stave model. Finally, Matsunaga et al. [66] observed that monolayers of 1,2-dihexanoyl-sn-glycero-3-phosphocholine (DHPC) formed on alkanethiol-modified gold surfaces in a buffer solution can suffer phase transitions driven by potential electrochemical control. The monolayers can shift among fluidic-I, striped fluidic-II, and grainy phases.

### 3.3. Proteins

Proteins are of paramount importance for living systems because their function provides the structural stiffness and rigidity to define the distinct shape of each living being. Proteins also have key roles: catalyzing cellular chemical reactions in the immune system, signaling, and signal transduction. Proteins are also valuable for biotechnological applications. Protein structure is determined by X-ray crystallography or nuclear magnetic resonance (NMR), which are considered the gold standard because they bring three-dimensional information at high resolution. STM will never compete with these techniques, but it can be a complementary tool. For example, some proteins cannot benefit from X-ray because they cannot form crystals of good quality for X-ray diffraction. Some proteins are sensitive proteins that degrade upon radiation exposure. Proteins such as membrane proteins frequently produce only tiny crystals [67]. Additionally, the information obtained only refers to the average configuration of all the molecules forming the crystal. Thus, information about structural changes at the level of individual molecules is prohibitive. In the case of NMR, it requires higher concentrations of biomolecules than X-ray crystallography. Further, the samples need to be stable for long periods since the data acquisition can take weeks [58]. In the case of systems that integrate proteins with electronic elements for different technological applications, STM can provide an accurate morphological characterization of single adsorbed proteins, which can be problematic for X-ray and MNR [68].

Among the first justifications to analyze proteins with STM was to explore alternatives for the structural analysis. Edstrom et al. [69] reported the direct observation of phosphorylase kinase and phosphorylase b by STM using HOPG as substrate. The shape and subunit organization of proteins were discernible, the dimensions matched with previous data of X-ray crystallography, and the resolution was better than the previously obtained by electron microscopy. Years later, Miles et al. [70] analyzed the structure of the high molecular weight (HMW) subunit of wheat gluten protein. Arakawa et al. [71] analyzed the turtle α-macroglobulin deposited in HOPG and the analysis was performed under air conditions, showing that STM can resolve the structure of proteins at submolecular resolution with a comparable or better resolution to the obtained by TEM.

**Figure 4:**
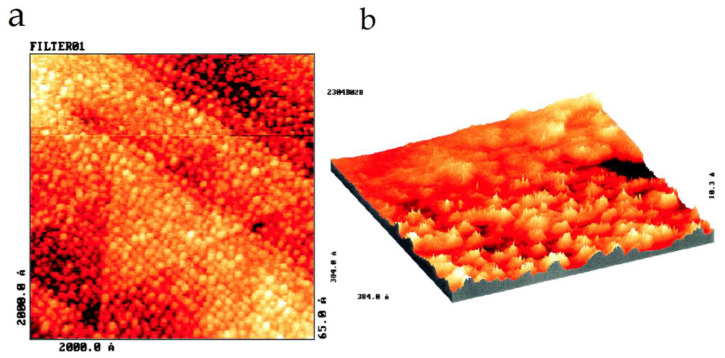
STM images of protein azurin deposited on Au(111). The spots represent individual azurin molecules (**a**). The 3D image shows the proteins exhibited a high central contrast that was interpreted as an area of high tunneling conductivity (**b**). STM analysis was performed in liquid. Reprinted with permission from Ref. [77]. Copyright (1999), National Academy of Sciences.

After these first studies, several proteins were analyzed underwater or in air conditions, including cytochrome c551 [72], glucose oxidase [73], hemoglobin [74], catalase [75], cytochrome c [75], azurin [76,77], rubredoxin [78], and albumin [79]. The advantage of STM for the analysis of one kind of enzyme named metalloenzymes was tested; these enzymes have metal ions that play key roles in their biological activity [80]. The blue copper protein azurin is a metalloenzyme and contains one copper atom in its structure. This protein was deposited on Au(111) and analyzed by STM in water conditions by Friis et al. [77]. STM images exhibited a central area of high contrast, with a 2- to 4-fold current enhancement compared with peripheral parts, which was assigned as the copper atom (Figure 4).

Davis et al. [76] analyzed the protein Zn7 metallothionein on gold and liquid conditions. This protein contains seven zinc atoms, arranged in two clusters, one containing four metal atoms and the other with three metal atoms. It is thought that dry samples are less conductive. Interestingly, STM images showed a current enhancement on the expected positions of the metal centers. These studies demonstrated that STM can be used to analyze the structure of proteins and their electronic properties. In contrast, Contera et al. [81,82,83] compared the metalloenzyme pseudoazurin (pAz) in its holo and apo forms in air conditions. The holo form contains copper, while the apo form does not. In this analysis, the authors did not observe any influence of the Cu ions in the holo form, and no significant difference was observed between the two forms. However, several reports have stated that water can play an essential role in forming STM images. Studies of dehydrated proteins have shown that the images of these samples display a shallow image contrast, which improves when the samples are hydrated [84].

Recently, several globular proteins such as streptavidin, antibodies, and EGFR kinase domain as monomers and dimers were studied by STM in solution [85,86]. STM images showed the samples have high mobility, and the proteins adopted different orientations. In some cases, the orientations resembled the reported crystal structures. The high mobility was explained because of the experimental conditions; it was performed in solution. The samples were not fixed to the substrate; thus, the proteins could adopt different orientations and move laterally and vertically.

**Figure 5:**
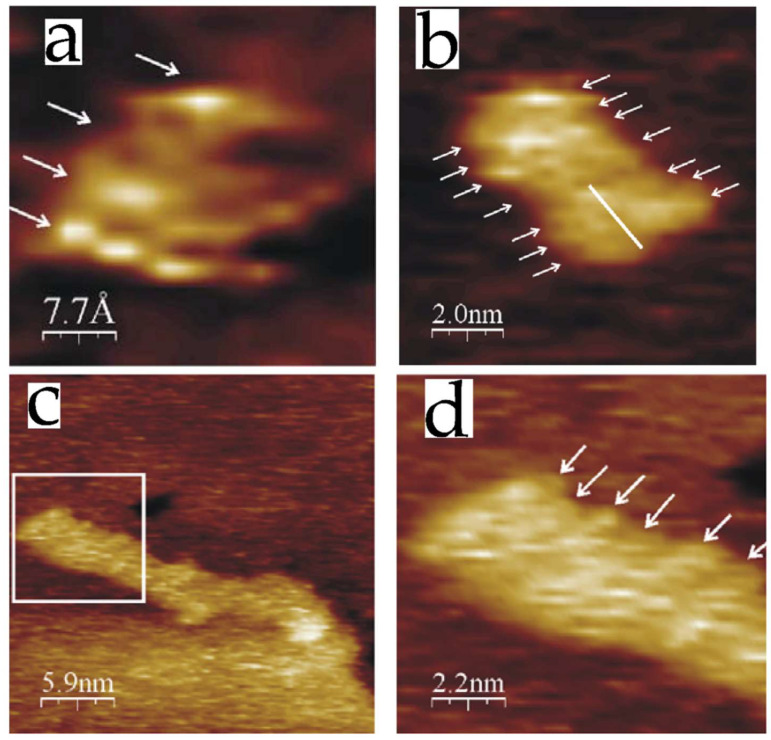
STM images of monomers, dimers, and oligomers of Aβ1–40 on atomically flat gold surfaces. Monomers contain three or four parallel strands (arrows) (**a**). Dimers are constituted by two monomers (**b**). Oligomers resulted from the fusion of monomers or dimers (**c**), the arrows are indicating the parallel strands (**d**). Reprinted with permission from Ref. [89]. Copyright (2006), Elsevier.

**Figure 6:**
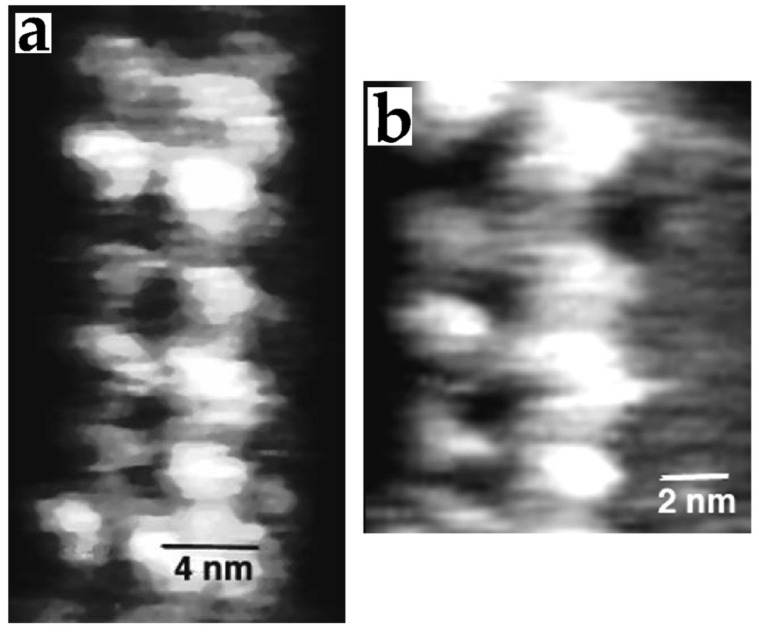
STM images of unfixed microtubules deposited on ITO surface. Typical images of microtubules (**a**,**b**). The images represent a portion of a microtubule, in which the arrangement of the tubulin proteins is slightly revealed. Reprinted with permission from Ref. [12]. Copyright (1994), The Company of Biologists.

STM was used to analyze proteins of medical importance. Aggregated amyloid β (Aβ) peptides in plaques and tangles are the histopathological hallmarks of Alzheimer’s disease. The aggregation pathway of precursor amyloid peptides to mature fibrils is of great value. There is an emerging consensus that soluble oligomeric species such as soluble dimers, trimers, and small oligomeric aggregates but not monomers are more pathogenic species in Alzheimer’s than insoluble mature fibrils and dense fibril meshes. Thus, understanding the structural details of oligomers is an essential first step toward elucidating the aggregation pathway involved in plaques. Detailed structural information obtained by STM can help rational design therapeutic tools and methods for diagnostics. Studies by STM have been conducted to characterize the morphology of β-amyloid fibrils [87,88] and oligomers [89,90] at the submolecular level of resolution. Notably, Losic et al. [89] provided high-resolution STM images of Aβ monomers, dimers, and oligomers for the first time (Figure 5). Remarkably, with the high level of resolution, they suggested a possible mechanism for oligomerization, which consisted of the sequential addition of monomers to form dimers and oligomers. Forman et al., in 2013, analyzed amyloid fibers decorated with monomers of cytochrome b562 and the monomeric multi-haem protein, GSU1996. These structures were engineered as conducting fibers for new devices. STM images allowed the visualization of the amyloid fibers as dark stripes and the attached proteins as conducting peaks [91]. STM is not restricted to small proteins [11]; complexes of proteins such as collagen I [92] and microtubules (Figure 6) [12] were studied.

**Figure 7:**
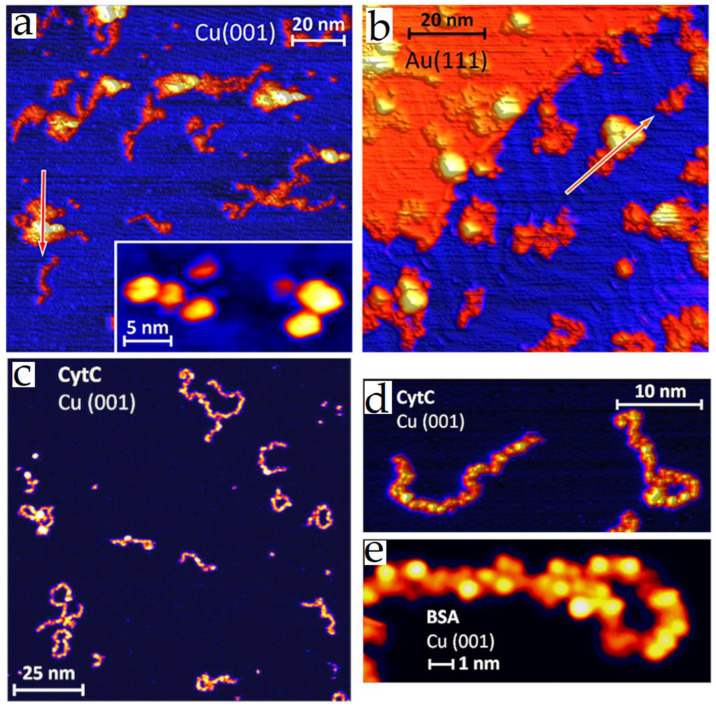
STM images of CytC deposited by the ES-IBD method onto copper (**a**,**c**,**d**) and gold surfaces (**b**). Folded proteins displayed a globular structure and unfolded proteins displayed an extended conformation, and the observed lobes on extended proteins were interpreted as amino acids (**d**,**e**), BSA was used as reference (**e**). Reprinted with permission from Ref. [93]. Copyright (2020), American Chemical Society.

Recently Deng et al. [93] utilized electrospray ion beam deposition (ES-IBD) to deposit cytochrome C proteins in different substrates such as Cu(001), Au(111), and BN/Rh(111) substrates. With this approach, they folded or unfolded proteins on the substrates controlling the pH of the electrospray solution and filtering the charge states (unfolded proteins were found mainly as high-charge state ions (z > +10). In contrast, folded proteins were found as low-charge states (z < +8)). Notably, STM images of folded proteins displayed a globular structure (Figure 7). Meanwhile, unfolded proteins were observed as extended polymer strands. STM images of high resolution revealed internal lobes structures inferred as groups of amino acids. The reports indicated can give direct information for the structure and organization of these types of assemblies and complement the classical methods of electron microscopy and X-ray diffraction.

### 3.4. Carbohydrates

Carbohydrates play various biological functions, including a source of chemical energy, energy storage, communication messenger, a structural component of cell membranes, and functions as the key structural component in plants [94]. Carbohydrates can be found in nature as simple molecules (monosaccharides), joined together (disaccharides, oligosaccharides, and polysaccharides), or as components of biopolymers (glycolipids, glycoproteins) [95]. The analysis of carbohydrates by STM can be of great value since compounds with molecular weights ranging from 1000 to 5000 are difficult to crystallize [94].

**Figure 8:**
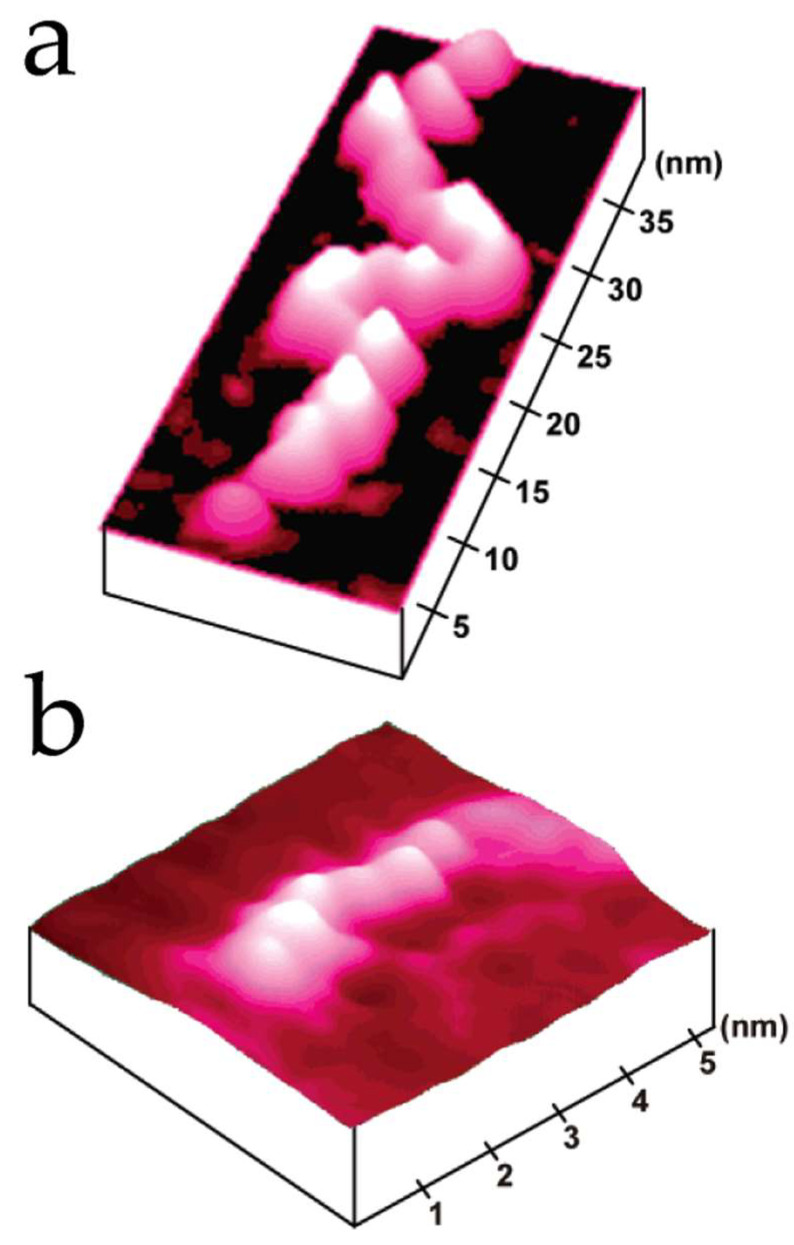
STM images of molecular necklaces, where (**a**) is a necklace of 22 α-CyDs and (**b**) one of 15 α-CyDs. Reprinted with permission from Ref. [100]. Copyright (2003), American Chemical Society.

**Figure 9:**
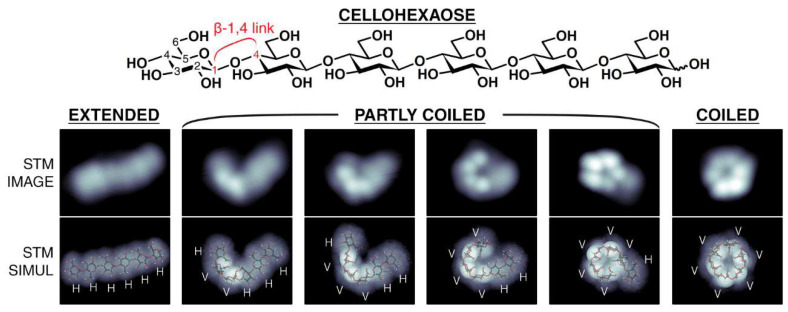
STM image and STM simulation of cellohexaose on Cu(100). The deposition parameters’ control permitted the transition conformers between “extended” and “coiled” forms of cellohexaose. Reprinted with permission from Ref. [105]. Copyright (2020), American Chemical Society.

Additionally, many carbohydrates are epimers, anomers, and regioisomers, and are challenging to identify without additional chemical steps. As an alternative, the use of STM was explored to analyze carbohydrates. Among the first studies were the analysis of β-cyclodextrin [18,96], cellulose [97], glycogen [98], and polysaccharides [99]. Miyake et al. [100] analyzed the structure of the cyclodextrin (CyD) necklace, which consist of cyclodextrin units threaded onto a poly(ethylene glycol) (PEG) chain. Notably, they analyzed one necklace of 22 α-CyDs and one of 15 α-CyDs on HOPG and MoS_2_. They reported that the molecular necklaces were stable and observed during the analysis (Figure 8a). In the case of 22 α-CyDs, cyclodextrins were observed separately on the PEG chain, and in 15 α-CyD, the molecules were observed closely packed (Figure 8b). Their results found that nearly 20% of the cyclodextrins were in a head-to-tail conformation. A few years after, Jia et al. [101] analyzed a cyclodextrin necklace based on cyclodextrin units threaded onto biodegradable and thermoresponsive polyurethanes derived from bile acids. In their STM images, the cyclodextrins were observed as aligned bright spots on the Au(111) substrates. Abb et al., in 2019 [102,103] analyzed the disaccharides sucrose and trehalose. They employed soft-landing electrospray ion beam deposition (ES-IBD) to deposit the molecules on Cu(100) substrates. In their analysis, sucrose was observed as elongated double lobes with dimensions of 1.0 ± 0.2 nm in length and 0.5 nm width; these dimensions fit the expected size of a sucrose molecule. Moreover, the trehalose molecules showed a globular structure with 0.9 ± 0.1 nm in length and 0.6 ± 0.1 nm in width. These dimensions agree well with the expected size of a disaccharide molecule. More recently, complex oligosaccharides were studied by Wu et al. [104] in 2020, and it was found that STM was able to visualize the connectivity in glycans and the resolution allowed the discrimination between regioisomers. In this study, ES-IBD was also used to deposit the samples. The same group extended their analysis and, employing soft molecule–surface collision, analyzed oligosaccharides’ conformation states; mainly, they found that cellohexaose can be found in diverse conformation states: extended conformation, partly coiled conformation, or a coiled conformation (Figure 9). Additionally, they found that the coiled population was the primary gas-phase conformer, and their presence can be controlled by tuning the collision energy [105].

Very little work was done for the analysis of carbohydrates. The group of Klaus Kern [102,103,105,106] started to explore a new method for sample deposition, opening the possibility to analyze complex carbohydrates (e.g., glycosaminoglycans, glycolipids, and glycoproteins). The STM can be valuable to carbohydrates’ inherent polydispersity and microheterogeneity.

## 4. Relevance of STM Imaging in Biological Applications

Imaging single biomolecules have been a long-standing ambition to advance various fields, such as structural biology, biophysics, nanotechnology, and medicine. Exceptionally, direct STM imaging of single biomolecules can be helpful to address medical problems that require structural and dynamic information of biomolecules, mainly those that adopt heterogeneous and non-crystalline structures or for those which crystals cannot be obtained. For example, amyloid fibrils are a central pathological feature of Alzheimer’s disease and other neurodegenerative diseases [107]. The characterization of oligomers preceding the formation of well-defined fibrils and the fibrils themselves are of particular interest because the understanding of the structure and dynamic assembly of fibrils can help to understand the abnormal assembly and could be used for the rational design of therapeutic agents for their prevention or disaggregation. Structural studies have shown it is generally not possible to use conventional methods such as crystallography or solution nuclear magnetic resonance since the fibers are insoluble and the oligomers are polymorphic. In this regard, STM has been used to analyze oligomers and provided high-resolution STM images of Aβ monomers, dimers, and oligomers [89]. STM has proved its utility in understanding the processes of fibril formation.

STM also offers advantages over conventional techniques such as X-ray and electron microscope. STM can be used to study the electronic properties of proteins, particularly those involved in the conversion of energy in organism autotrophs and heterotrophs. As mentioned in Section 3.3, metalloproteins are enzymes with metal ions that play a crucial role in photosynthesis, respiration, and other biological processes where the production of glucose and ATP is vital. Metalloproteins are very efficiently shuttling single electrons over long distances and quickly among the active sites of biological partners [108]. STM has been used not just for imaging of metalloproteins but also for analyzing electron transport across these proteins in air and under aqueous conditions with a high spatial resolution. EC-STM has been used to study small proteins, cytb_562_, cyt c, and azurin, multiprotein complexes as photosystems, and various metalloproteins [109]. Remarkably, STM has shown to be valuable for analyzing proteins at a single-biomolecule level.

STM in biological applications can be related to nanotechnology. Nanoscale structures and materials (nanoparticles, nanowires, nanofibers, nanotubes) have been explored in many biological applications (biosensing, biological separation, molecular imaging, anticancer therapy) because their novel properties and functions differ drastically from their bulk counterparts [110,111]. This is the field of nanobiotechnology, which combines engineering and molecular biology, leading to novel multifunctional devices and systems for biological analysis with better sensitivity, specificity, and a higher recognition rate. The high demand and production of that new class of devices and systems will require characterization techniques to be versatile and low cost.

An exciting application of biological nanoparticles is using RNA particles for tumor diagnosis. Shu et al. [112] prepared RNA nanoparticles to target cancer exclusively in vivo without accumulating normal organs and tissues. The advantage of this application is that these miniaturized fluorescent nanoparticles can be quickly taken up by cells through endocytosis and subsequently used for site-specific intracellular measurements. The use of STM for imaging those particles can help analyze large biomolecules in real-time. However, breakthroughs in sample preparation techniques or STM instrumentation may be a conditional factor for this application.

Dumont and Prakash [1] examined the hierarchical thread in which single molecules give rise to a complex and dynamic structure at the cellular scale. Probing the mechanics of complex macromolecular assemblies inside cells has been challenging for a long time. Such mechanics require quantifying temporal dynamics, precise architectural parameters, active force generation processes, and their relationship [113]. In this regard, STM/STS may also be essential to measure electronic states and thus characterize individual molecules as a response to modifications to their chemical structure.

Recently, Xia and Kanchanawong reviewed open questions about the cell adhesions at the nanoscale level to grasp the inner logic of cellular decision-making [114]. The need to characterize forces and imaging cellular ultrastructure is only reachable through advanced techniques such as super-resolution fluorescence microscopy coupled to electron microscopes [115]. STM/STS might evolve in advanced techniques to provide a three-dimensional profile of the surface and local information at the contact point between two different cell regions in a similar fashion to those measurements for semiconductor heterojunctions [116].

Integrating biomolecules with synthetic materials can direct self-assembly, catalysis, and other specific properties such as biorecognition. This can be exploited when using biohybrid systems as electronic devices for diagnostics, health care, drug screening, or environmental monitoring applications [117]. For those applications, integrating biomolecules into inorganic surfaces is of great importance since the distribution and orientation of biomolecules on surfaces can affect their functionality. Surface plasmon resonance (SPR) sensors [118] are thin-film refractometers that measure changes in the refractive index occurring at the surface of a metal film supporting a surface plasmon. Various types of biorecognition elements have been employed in affinity SPR biosensors. Single-chain antibodies fragments remain by far the most frequently used biorecognition element. Another type of biorecognition element that has been employed in SPR sensors is peptides and aptamers. These biorecognition elements are commonly immobilized on the sensor surface. The surface chemistry must be designed to enable immobilization of a sufficient number without affecting their biological activity [119]. Therefore, SPR sensors still require high-cost investment, components, and operation, leading to unaffordability for implementation in a low-cost point of care or laboratories [120]. In this regard, STM can help to analyze and characterize the SPR sensors since STM has proven to be a powerful tool for analyzing biomolecules adsorbed on surfaces [68].

## 5. Perspective

STM has proven to be an invaluable tool for studying conductive surfaces throughout the last four decades. The incursion of novel substrates and new deposition techniques helped to improve the capability to resolve structures at the atomic level, allowing the study of complex phenomena such as molecular bonding and hybridization states [121], in-situ chemical reactions for molecular engineering [122], or single-molecule junctions [123], to mention a few applications. These advances drive STM expansion in all directions and help conceptualize other instruments such as the AFM [124] or the scanning ion conductance microscope [125] that also allow the observation of biological samples.

The most cited article featuring the capabilities of STM to resolve sub-nanometer structures was focused on the analysis of carbon nanotubes (CNTs). In 1998, Odom et al., [126] were able to locate and resolve the hexagonal-ring structure of the CNTs walls. These results represented a significant implication to determine the structure of such nanosystems, which were promptly compared with theoretical models. At that time when STM imagined CNTs, this technique was still a young child [127] that required the accompaniment of other mature techniques such as ultra-high vacuum systems, controlled atmospheres, sputtering, annealing, and cooling systems (<100 K). Hence, STM owned fundamental implications in the physics and surface-science communities.

STM grew up in the following years but was accompanied by scanning tunneling spectroscopy (STS). The couple STM and STS correlate well the structure and the density of electric states that are of paramount importance for electronic applications. This fact fits with the original prospect from their STM inventors, Binning and Roher [128], which was to use STM as a Feynman molecular engine [129] to handle atoms and carry out small force measurements down to those between individual atoms. High-resolution STM/STS experiments are probably the proof for that expectation, as shown in the observational measurements of different types of nanostructures with variable composition and bandgap [130].

In the last decade, the emphasis in STM studies was shifted from the research of surface topography to surface chemistry [122] and surface dynamics [131]. In the case of STM microscopists, a social differentiation within the SPM community made it an attractive opportunity to focus on nanoscience as the most attractive proposition for research. Therefore, the atomic resolution capability in STM was taken as what makes it unique to use as a tool in nanotechnology. The STM research was pushed forward with the participation of surface scientists and nanotechnologists while it was moving away from bio-applications.

In this way, biologists found the whole language of electron tunneling obscure and unhelpful in interpreting these strange images. At the same time, surface scientists identified biological systems as “dirty” and ill-defined [132]. Today, the complexity in STM imaging interpretation has motivated experimental and theoretical groups to understand the origins of image contrast [16] as the basis for interpreting images. In addition, STM is a technique requiring specialized operators to optimize imaging parameters during sample analysis. Their ability to recognize and identify structures may help spread the interest in using STM in other applications than surface phenomena. The current STM experiments in biological systems may require feedback from atomistic simulations to reproduce the key features of the imagined structures [133]. Therefore, STM for imaging biological structures can be an opportunity to train researchers who exceptionally can endorse the instrument attending the sample preparation and imaging interpretation.

In perspective, STM applications for biomedicine are still an elusive goal, albeit not necessarily unreachable. Our experience says that the electron microscope is preferable in nanoscale research. STM can require several fiddling days to obtain meaningful images; in comparison, a few hours are needed when working with electron microscopes [79]. However, our first option is to use STM when studying biological structures attached to nanostructures. In general, the recent STM studies are clear evidence that the era of this tool is not at its end but heading towards new ways other than applications by which molecular systems can be manipulated and studied. STM imaging, computational chemistry methods, sample deposition, and UHV techniques are pivotal in characterizing biological structures. A continuous improvement in the physical features of substrates is also profitable in both academic and commercial fields [134]. The STM research is still an entire science research ecosystem; it is open for practitioners from various disciplines interested in biology, technology, and instrumentation. History tells us that microscopy played a crucial role in the growth of nanotechnology in which nanostructures are frozen in space and time. STM can assist studies about dynamic biological systems with relevance for nanobiotechnology applications.
